# Midterm performance of decellularized equine pericardium in congenital heart surgery

**DOI:** 10.1093/icvts/ivac269

**Published:** 2022-11-07

**Authors:** Viktoria H M Weixler, Kira Kuschnerus, Olga Romanchenko, Stanislav Ovroutski, Mi-Young Cho, Felix Berger, Matthias Sigler, Nicodème Sinzobahamvya, Joachim Photiadis, Peter Murin

**Affiliations:** Department of Congenital Heart Surgery - Pediatric Heart Surgery, German Heart Center Berlin, Berlin, Germany; DZHK (German Centre for Cardiovascular Research), partner site Berlin, Germany; Department of Congenital Heart Surgery - Pediatric Heart Surgery, German Heart Center Berlin, Berlin, Germany; Department of Congenital Heart Surgery - Pediatric Heart Surgery, German Heart Center Berlin, Berlin, Germany; Department of Congenital Heart Disease - Pediatric Cardiology, German Heart Center Berlin, Berlin, Germany; Department of Congenital Heart Surgery - Pediatric Heart Surgery, German Heart Center Berlin, Berlin, Germany; DZHK (German Centre for Cardiovascular Research), partner site Berlin, Germany; Department of Congenital Heart Disease - Pediatric Cardiology, German Heart Center Berlin, Berlin, Germany; Department of Pediatric Cardiology and Intensive Care Medicine, Georg-August University Göttingen, Göttingen, Germany; Department of Congenital Heart Surgery - Pediatric Heart Surgery, German Heart Center Berlin, Berlin, Germany; Department of Congenital Heart Surgery - Pediatric Heart Surgery, German Heart Center Berlin, Berlin, Germany; Department of Congenital Heart Surgery - Pediatric Heart Surgery, German Heart Center Berlin, Berlin, Germany

**Keywords:** Decellularized equine pericardium, Congenital heart surgery, Pericardial patch

## Abstract

**Objective:**

The goal was to report the midterm performance of decellularized equine pericardium used for repair of various congenital heart defects in the paediatric population.

**Methods:**

A retrospective review of all patients undergoing patch implants between 2016 and 2020 was performed. Patch quality, surgical handling, haemostasis and early patch-related complications were studied in all patients. Midterm performance was observed in patients with ≥12 months follow-up and an intact patch at discharge (without reoperation/stent implant).

**Results:**

A total of 201 patients with a median age of 2.5 years [interquartile range: 0.6-6.5] underwent 207 procedures at 314 implant locations. The patch was used in the following numbers/locations: 171 for pulmonary artery (PA) augmentation, 36 for aortic repair, 35 for septal defect closure, 22 for valvular repair and 50 at other locations. Early/30-day mortality was 6.5%. Early patch-related reoperations/stent implants occurred in 28 locations (8.9%). No patch-related complications were noted except for bleeding in 3 locations (1%). Follow-up for ≥ 12 months was available for 132 patients/200 locations. During a median follow-up of 29.7 months [interquartile range: 20.7-38.3], 53 patch-related reoperations/catheter reinterventions occurred (26.5%), with the majority in the PA position (88.7%, 47/53). Overall 12- and 24-months freedom from patch-related reoperation/catheter reintervention per location was 91.5% (95% confidence interval: 86.7%-94.6%) and 85.2% (95% confidence interval: 78.9%-89.6%), respectively.

**Conclusion:**

Decellularized equine pericardium used for repair of various congenital heart defects showed acceptable midterm performance. The range of reoperation/reintervention rates was similar to those observed with other xenogeneic materials in previously reported articles, occurring most frequently after PA augmentation.

**Figure:**
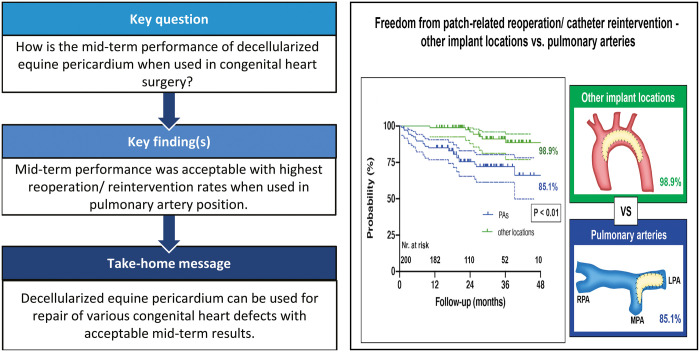

## INTRODUCTION

Repair of congenital heart defects often requires biological or synthetic patch materials for reconstruction of vascular structures or intracardiac defects. Ideally, autologous tissue is used because it is readily available and because it exhibits superior calcification resistance compared to other materials [1]. In the case of redo operations, however, because autologous pericardial patch material is unavailable, several alternatives such as Dacron (polyethylene terephthalate), Gore-Tex (polytetrafluoroethylene), glutaraldehyde-preserved bovine pericardium and allografts are used. Some of the shortcomings of these patch materials are inability to grow, shrinkage, risk of infection, aneurysm formation, thromboembolism and degeneration/calcification [2–7]. When it comes to preservation/fixation, glutaraldehyde especially has been critically evaluated due to its cytotoxicity and therefore its limitations for long-term performance [8]. Dye-mediated photo-oxidation and decellularization are alternative methods to the traditional glutaraldehyde fixation, which promise to improve tissue immune-compatibility and at the same time preserve structure and biomechanical properties [3, 9–11]. Decellularized equine pericardium (Matrix Patch, Auto Tissue Berlin GmbH, Berlin, Germany) is a tissue-engineered, cell-free equine-derived pericardium processed through a glutaraldehyde-free technique, which sterilizes and reduces the residual DNA content to a minimum so that transmission of pathological micro-organisms is impossible. Additionally, the company promises technical feasibility and amelioration of calcification. Its performance in the paediatric population, however, is unknown. Our group recently published encouraging early results of its use in the pulmonary artery position [12]. Because of the increasing complexity and diversity of developments in the field of paediatric cardiac surgery, a patch material with the following characteristics is needed: high biocompatibility and resistance to infections, calcifications, thromboembolism and degeneration/sustainability towards the pulmonary and systemic circulation [13]. The systemic circulation in particular remains challenging when it comes to patch durability [4]. In a large-animal study in sheep, decellularized equine pericardium used in the systemic circulation showed promising early results [14]. However, reports on its use in humans, especially mid-/long-term results, are lacking [15]. Therefore, our goal was to report our experience with this patch when used in various anatomical locations under both pulmonary and systemic pressure conditions and to report early performance as well as midterm durability.

## PATIENTS AND METHODS

### Ethics Statement

Ethical approval was given by the ethics committee of the Charité on 4 June 2020 (EA2/081/20). Informed consent for data analysis was waived because of anonymity and the retrospective design of the study. In child participants, formal consent was obtained from the parent/guardians. Due to an available Conformité Européenne mark in Europe, no consent was needed to implant the patch.

### Study design and population

A retrospective observational study was conducted. At the time of the implant, the patch was certified for use in vessel augmentation, heart valve reconstruction and closure of intracardiac septal defects. All patch implants in patients undergoing congenital heart surgery at our institution between 09/2016 and 12/2020 were included.

### Study end points

Primary study end points were patch-related early/late mortality and patch-related reoperations/catheter reinterventions due to bleeding from patch site, patch insufficiency/dilatation or early restenosis.

Any death occurring during the same admission or during 30 postoperative days was defined as early death. A death was defined as patch-related when it occurred in direct association with the implanted patch.

Reinterventions were considered early when they occurred before discharge. Among other parameters, major postoperative complications (renal failure requiring dialysis, heart block requiring a pacemaker implant, persistent neurologic impairment, postoperative mechanical circulatory support, phrenic nerve injury and unplanned reoperation) and total stays in the intensive care unit and the hospital were assessed [16].

Secondary end points such as intraoperative handling, patch-related complications (bleeding, rupture, excessive dilatation, thrombosis, endocarditis and calcification at the patch location leading to loss of function) and non-patch-related early/late deaths were studied.

Intraoperative handling of decellularized equine patch implants was compared to the surgical experience with autologous pericardium and was rated in 3 categories: intact quality/smoothness of patch material, surgical feasibility/easy patch handling and haemostasis. Each category was rated from 0 to 3 (0=bad; 1=acceptable; 2= good; 3=excellent) and added to a total score of 0 to 9, which was calculated by the individual surgeon.

### Follow-up

**Figure 1:**
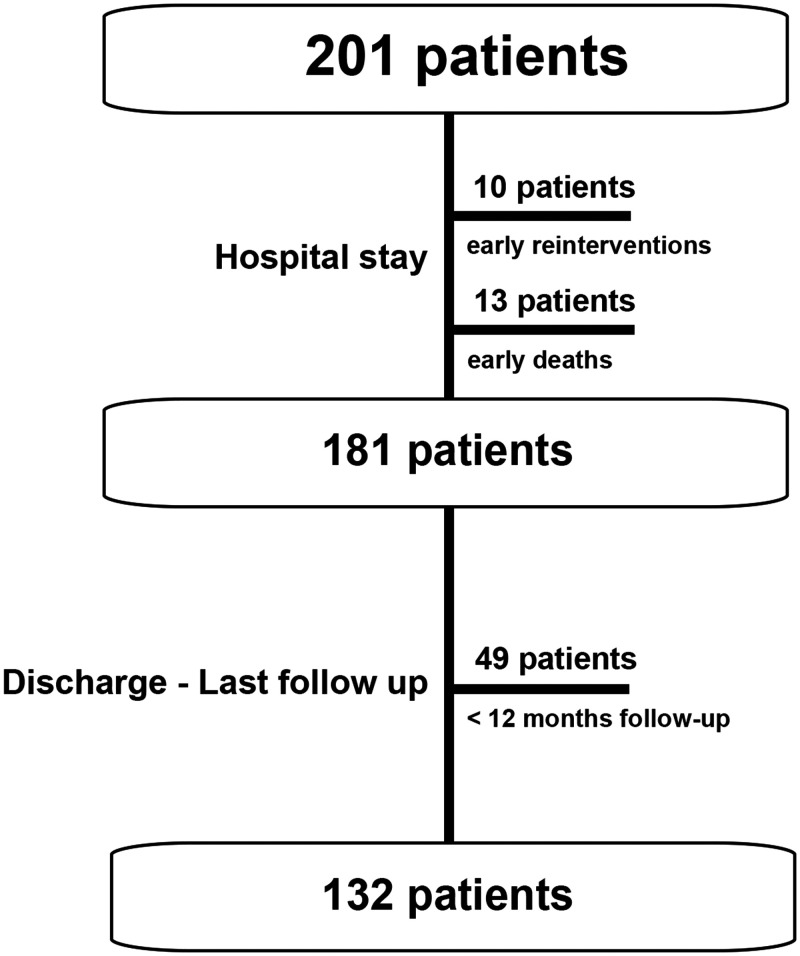
Depiction of the study population. Starting with an initial study population of 201 patients, 23 patients were excluded from further follow-up due to early reinterventions (stent implant or surgery) prior to discharge and/or early death. Another 49 patients had to be excluded due to a follow-up < 12 months. The remaining 132 patients were followed until the last visit.

Survival, intraoperative handling, early patch-related complications and patch-related reoperations/catheter reinterventions before discharge were studied in the entire study population. Midterm durability and patch performance were studied in patients with ≥12 months follow-up and with an intact patch location at discharge (no early reoperation or stent implant before discharge). Medical records including preoperative data, surgical notes and postoperative clinical, echocardiographic, angiographic and outpatient reports were searched for this information. We purposely chose to exclude patients with < 12 months follow-up because the goal of this study was to evaluate midterm patch durability/performance. Additionally, the goal was to distinguish between “early patch/technical failure” and “late failure/patch remodelling”. For this purpose, patients with less than 12 months of follow-up were excluded from midterm patch performance analysis. The study population is displayed in Fig. 1.

### Surgical procedures

The range of applications varied from septal defect closure (atrial/ventricular septal defect), pulmonary artery augmentation [right/left/main pulmonary artery (PA)], right ventricular outflow tract enlargement, pulmonary or systemic vein enlargement (superior/inferior vena cava, Glenn procedure), left ventricular outflow tract repair (aortic root, ascending aorta, aortic arch), valvular/leaflet repair (aortic/pulmonary/mitral/tricuspid valve) and others (e.g. sinus of Valsalva repair, coronary artery repair). Surgical complexity of the primary procedure was stratified based on the Society of Thoracic Surgeons–European Association for Cardio-thoracic Surgery) mortality scores and categories [17].

The soft and pliable structure of the patch especially allowed for the delicate repair of septal defects and pulmonary arteries/veins. Because we had just started using this patch material in the systemic circulation and/or complex valvular reconstructions, we were restrictive in its application. In general, great effort was made to create a 3-dimensional geometric patch by avoiding tissue redundancy and appropriate patch tailoring. For easier handling, attachment of the patch on a piece of cardboard using haemoclips was performed (Table 1).

**Table 1:** Patient baseline and procedural characteristics

Characteristics	Values
**Patients**	N = 201
** Age at surgery** (years) median [IQR]	2.5 [0.6-6.5]
** Sex** (male) n (%)	107 (53.2)
** Weight at surgery** (kg) median [IQR]	12 [6.8-20.3]
** BSA** (m^2^) median [IQR])	0.6 [0.4-0.9]
** Diagnosis,** n (%)	
** **PA/PS/TOF	50 (24.9)
** **HLHS	27 (13.4)
** **DORV-d-TGA	26 (12.9)
** **AVSD	23 (11.4)
** **TAC	12 (5.9)
** **TA	9 (4.5)
** **cc-TGA	8 (3.9)
** **Aortic arch hypoplasia/IAA	7 (3.5)
** **Others	39 (19.4)
** Previous cardiac surgery,** n (%)	169 (84.1)
**Procedures**	N=207
** Primary procedure,** n (%)	
** **PA/RVOT enlargement/TAP	55 (26.6)
** **Glenn procedure	16 (7.7)
** **Norwood/DKS	15 (7.2)
** **DSO/Nikaidoh/double root translocation	11 (5.3)
** **Fontan operation	10 (4.8)
** **Mitral valve repair	10 (4.8)
** **Pulmonary vein repair	8 (3.9)
** **Aortic arch repair	7 (3.4)
** **VSD closure	7 (3.4)
** **ASD closure/PAPV repair	5 (2.4)
** **Others	63 (30.4)
** STAT score,** median [IQR]	1.4 [1.2-2.5]
** STAT mortality category,** I-V, n (%)	**I**: 25 (12.1)
	**II**: 50 (24.2)
	**III**: 44 (21.3)
	**IV**: 69 (33.3)
	**V**: 19 (9.2)
** Biventricular repair,** n (%)	125 (60.4)
** CPB time,** min, median [IQR]	196 [132.5-283.5]
** CC time,** min, median, [IQR]	65.5 [0-123.8]

Continuous data are presented as median, interquartile range [IQR]; categorical data are presented as n, %.

ASD: atrial septal defect; AVSD: atrioventricular septal defect; BSA: body surface area; cc-TGA: congenitally corrected transposition of the great arteries; DKS: Damus-Kaye-Stansel; DORV: double outlet right ventricle; DSO: double-switch operation; CC: cross-clamp; CPB: cardiopulmonary bypass; d-TGA: dextro-transposition of the great arteries; HLHS: hypoplastic left heart syndrome; IAA: interrupted aortic arch; IQR: interquartile range; PA: pulmonary atresia; PS: pulmonary stenosis; RVOT: right ventricular outflow tract; STAT: Society of Thoracic Surgeons–European Association for Cardiothoracic Surgery; TA: tricuspid atresia; TAC: truncus arteriosus communis; TAP: transannular patch; TOF: tetralogy of Fallot; VSD: ventricular septal defect.

### Statistical methods

Baseline/intraoperative outcome measures are presented as the median [interquartile range (IQR)] when continuous data and as number (%) of patients/procedures/locations when categorical data are compared using the Mann-Whitney U-test and the χ^2^ test. The data were analysed on a per-patient, per-procedure and per-implant location basis, accounting for multiple procedures and implant locations per patient. Survival and freedom from patch-related reoperations/catheter reintervention were analysed using Kaplan-Meier time-to-event models. Freedom from patch-related reoperations/catheter reinterventions (balloon dilatation and/or stent implantation) was further studied in a subgroup analysis comparing the implant location of the PAs versus all other implant locations. Binary logistic regression was used to predict risk factors for any type of reintervention (stent implant, surgery, balloon dilatation). The model selection strategy was to include clinically important/relevant confounders/covariates (age, pulmonary vs other implant locations, previous operations/interventions at the site of the patch implant). The 2 groups (PAs vs other locations) were compared using the log-rank (Mantel–Cox) test. Statistical analyses were performed using IBM-SPSS version 24.0 (IBM-SPSS Inc, Armonk, NY, USA) and GraphPad Prism version 8.4.0 (GraphPad Software, Inc., San Diego, CA, USA). *P*-values <0.05 were considered statistically significant.

## RESULTS

### Study population

A total of 201 patients with a median age of 2.5 years [IQR: 0.6-6.5] and a median weight of 12 kg [IQR: 6.8-20.3] underwent 207 procedures with 314 patch implant locations. The patch was used in the following numbers and locations: 171 for repair of PAs, 36 for repair of the aorta, 35 for repair of septal defects, 22 for valvular repair and 50 at other locations. The majority of the patients had prior cardiac procedures (84.1%). The procedures ranged from low-complexity cases such as atrial septal defect or ventricular septal defect closure up to Norwood and double switch operations. Almost half (42.5%) of all the procedures were ranked as high risk (Society of Thoracic Surgeons–European Association for Cardio-thoracic Surgery mortality risk score 4–5) with 82 single-ventricle procedures (39.6%) (Tables 1, 2*)*.

**Table 2:** Patch implantations per location

Implant location	N (%) Total = 314
**Aorta**	
Aortic root	18 (5.7)
Ascending aorta	4 (1.3)
Aortic arch	14 (4.5)
**Valves**	
Aortic valve	3 (0.9)
Pulmonary valve	6 (1.9)
Mitral valve	11 (3.5)
Tricuspid valve	2 (0.6)
**Systemic veins**	15 (4.7)
**Pulmonary veins**	6 (1.9)
**Pulmonary arteries/RVOT**	
MPA	18 (5.7)
LPA/RPA	153 (48.7)
TAP/RVOT	20 (6.3)
**RA**	7 (2.2)
**LA**	2 (0.6)
**ASD/PAPVD**	19 (6.1/)
**VSD**	16 (5.1)

Categorical data presented as n (%).

ASD: atrial septal defect; LA: left atrium; LPA: left pulmonary artery; MPA: main pulmonary artery; PAPVD: partial anomalous pulmonary venous drainage; RA: right atrium; RPA: right pulmonary artery; RVOT: right ventricular outflow tract; TAP: transannular patch repair; VSD: ventricular septal defect.

**Figure 2:**
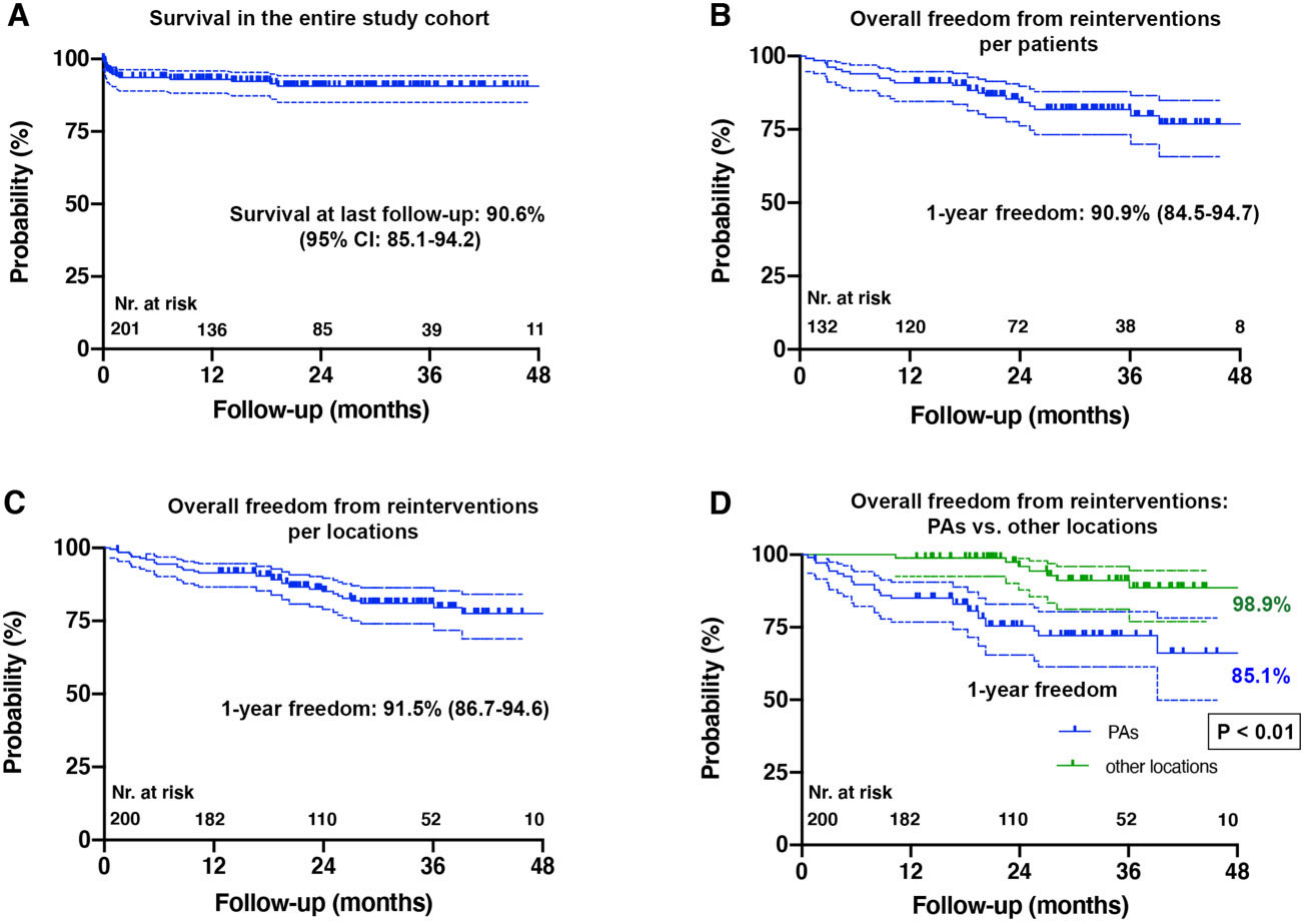
Survival and freedom from a patch-related reintervention (Kaplan-Meier time-to-event estimates). (**A**) Survival after a patch implant within the entire study population (N = 201). (**B**) Overall freedom from any kind of patch-related reoperation/catheter reintervention per remaining patients (N=132). (**C**) Overall freedom from any kind of patch-related reoperation/catheter reintervention per remaining locations (N=200). (**D**) Overall freedom from any kind of patch-related reoperation/catheter reintervention comparing PAs versus all other implant locations. Group comparison was performed using the log-rank (Mantel-Cox) test. CI: confidence interval; Nr.:number.

**Video.** Demonstration of the use of decellularized equine pericardium in various implant locations. **1.** Reconstruction of the right atrium lateral aspect after resection of a tumour in a newborn. **2.** Closure of ventricular septal defect during a double root translocation in an infant. **3.** Reconstruction of the right ventricular outflow tract in double valve homograft replacement in a child with truncus arteriosus communis (Supplementary material).

### Early outcome

Among the entire study population, the early/30-day mortality was 6.5% (13/201). No patch-related complications were noted except for bleeding from the implant site in 3 patients (1.5%, 3/201), in which augmentation of the right PA had been performed earlier. In all of the patients, extracorporeal membrane oxygenation assistance was necessary due to haemodynamic instability, and redo surgery for haemostasis without patch replacement was performed. According to the operative reports, the patch itself was described as intact in all cases. The reason for bleeding was classified as suture line insufficiency due to fragile native tissue. Two of these patients died early of multiorgan failure. Therefore, in 2/13 early deaths, even though they were not directly patch-related, the death can somehow be brought into a relationship with the patch implant.

Early reoperation/stent implant occurred in 10 patients (10/201; 4.9%) and 28 locations (28/314; 8.9%). The patch was intact at the time of discharge in 181 survivors (181/201; 90%) and in 286 locations (286/314; 91.1%). Early postoperative characteristics and major postoperative complications are summarized in Table 3.

**Table 3:** Early postoperative outcome

Variable	All patients (N=201)
**ICU stay** (days, median, [IQR])	4 [1-8]
**Total hospital stay** (days, median [IQR])	10 [7-19.3]
**Major complications,** n (%)	
Renal failure	11 (5.5)
Pacemaker implant	2 (1)
Neurologic complications	7 (3.5)
Paralyzed diaphragm	1 (0.5)
Mechanical assist device/VAD	6 (2.9)
Unplanned reoperation	33 (16.4)
**Early death** (n, %)	13 (6.5)
**Early patch-related complications** (n, %)	3 (1.5)
**Early reintervention at patch site** (surgical/catheter, n, %)	15 (7.5)
**Surgical** (n, %)	6 (2.9)
**Catheter-based**	9 (4.5)
Balloon dilatation (n, %)	5 (2.5)
Stent implant (n, %)	4 (2)
**Early survivors with intact patch at discharge** (n, %)	181 (90)

Continuous data presented as median and interquartile range [IQR]; categorical data presented as n, %.

ICU: intensive care unit; IQR: interquartile range; VAD: vascular assist device.

The total intraoperative handling score was ≥ 8 in 98.5% of the patients. Because bleeding occurred spontaneously after previous PA augmentation in 2 patients and after previous early balloon dilatation in the 1 patient, haemostasis was rated 0 in these patients, adding up to a total intraoperative handling score of 6.

### Follow-up

Survival in the entire study population was 92.6% (95% confidence interval [CI]: 87.7-95.5) at 12 and 91.8% (95% CI: 86.7-94.9) at 24 months after operations involving patch implants (Fig. 2A). One death occurred almost 2 years after discharge (1/132=0.8%) of multiorgan failure after a redo operation for mitral valve replacement, not patch-related. Clinical follow-up after discharge was available for 192 patients (overall data completeness=95.5%, 192/201) with a total of 9 patients who were lost to follow-up. The median follow-up time was 22.3 months [IQR: 10.8-36] for the entire study population. Follow-up ≥ 12 months was available for 132 patients and 200 locations with a median follow-up time of 29.7 months [IQR: 20.7-38.3]. During this follow-up time, a total of 53 patch-related reoperations/catheter reinterventions (balloon dilatations/stent implantations) occurred (26.5%; 53/200) (Tables 4, 5). The majority of patch-related reoperations/catheter reinterventions were observed after PA augmentation (88.7%; 47/53).

**Table 4:** Follow-up and patch-related reoperations/catheter reinterventions in early survivors with intact patch at discharge and ≥ 12 months of follow-up

Variable	Patients (N=132)
**Follow-up interval,** months, median [IQR]	29.7 [20.7-38.3]
**Total mortality after discharge,** n (%)	1 (0.8 )
**All reinterventions (catheter/surgical)**	N = 35
One reintervention, n (%)	23 (17.4 )
Two reinterventions, n (%)	9 (6.8 )
Three reinterventions, n (%)	2 (1.5 )
Four reinterventions, n (%)	1 (0.8 )
**Interval to reintervention (catheter/surgical)**	
First intervention, months, median [IQR]	10.3 [3.9-22.5]
Second intervention, months, median [IQR]	16.2 [11.1-22.6]
Third intervention, months, median [IQR]	30 [22-38]
Fourth intervention, months, median [IQR]	40.9 [40.9-40.9]
**Catheter reinterventions**	
Balloon dilatation, n (%)	13 (9.8%)
Stent implant, n (%)	8 (6.1%)
**Surgical reintervention, n (%)**	14 (10.6%)

Continuous data are presented as median, interquartile range [IQR]; categorical data are presented as n, %.

**Table 5:** Patch-related reoperations/catheter reinterventions in the remaining locations (intact at time of discharge and at ≥ 12 months follow-up)

Variable	Locations (N=200)
**All reinterventions (catheter/surgical)**	N = 53
One reintervention, n (%)	35 (17.5 )
Two reinterventions, n (%)	13 (6.5%)
Three reinterventions, n (%)	4 (2 )
Four reinterventions, n (%)	1 (0.5 )
**Catheter reinterventions**	
Balloon dilatation, n (%)	21 (10.5 )
Stent implant, n (%)	10 (5 )
**Surgical reintervention,** n (%)	22 (11 )
**Reason for reintervention,** n (%)	
Pulmonary artery stenosis	47 (88.7 )
Mitral valve regurgitation	2 (3.8 )
Ascending aorta aneurysm	1 (1.9 )
Pulmonary vein stenosis	1 (1.9 )
Biventricular repair	1 (0.9)
RVOTO	1 (1.9 )

Continuous data are presented as median, interquartile range [IQR]; categorical data are presented as n (%).

RVOT/O: right ventricular outflow tract/obstruction; TAP: transannular patch repair.

Overall freedom from patch-related reoperation/catheter reintervention (balloon dilatation and/or a stent implant) per patient, 12/24 months after initial surgery was 90.9% (95% CI: 84.5-94.7)/84.2% (95% CI: 76.2-89.7), respectively (Fig. 2B).

Overall freedom from patch-related reoperation/catheter reintervention (balloon dilatation and/or stent implantation) per location, 12/24 months after initial surgery was 91.5% (95% CI: 86.7%-94.6%)/85.2% (95% CI: 78.9%-89.6%), respectively (Fig. 2C).

### Risk factor/subgroup analysis

Binary logistic regression analysis revealed that the PA patch implant site was the only independent risk factor (OR=2.4, 95% CI: 0.9-6.9, *P*<0.028) for any type of reintervention (stent implant, surgery and balloon dilatation) in the follow-up period.

Freedom from patch-related reoperation/catheter reintervention (balloon dilatation and/or stent implant) 12/24 months after initial surgery was 85.1% (95% CI: 76.8–90.6)/75.5% (95% CI: 65.5-82.9) in the PA versus 98.9% (95% CI: 92.5-99.8)/97.5% (95% CI: 90.2-99.4) in other implant locations. The difference between the 2 groups was significant (*P*<0.001) (Fig. 2D).

## DISCUSSION

Children and adults with congenital heart disease represent a growing population requiring repeated complex operations. Because autologous pericardium is scarce in these circumstances, different xenogeneic patch materials have been proposed [3–5]. We initially started to use decellularized equine pericardium for PA augmentation with encouraging early results [12]. Even though the patch has the Conformité Européenne mark for the repair of septal defects and reconstruction of valvular/vascular structures, reports on its use are lacking [18, 19]. Thus, the performance/durability of decellularized pericardium in congenital heart surgery is unknown. Our goal was to complete the picture by studying all patients who received patch implants during repair of various congenital heart defects. Based on our institutional experience with different cell-free patch materials, we were cautious with the use of decellularized equine pericardium in systemic arterial and/or valvular locations [20].

When looking at early/late mortality in our study population, we were able to report satisfying results (early/late mortality: 6.5/0.8%), considering that the majority were redo operations (84%), with almost half of the procedures rated as high-risk (42.5%) and 40% being single-ventricle repairs. Our mortality rates were similar to those of previous groups reporting repair of congenital heart defects using other patch materials [3–5, 21]. In our cohort, 2 early deaths occurred in association with patch implants; in these patients, bleeding from the patch site led to reoperation and ultimately to multiorgan failure. However, in the operative reports, no patch rupture/patch-related tear/source of bleeding was described. Therefore, we concluded that major bleeding in the setting of a complex diagnosis and a high-risk procedure was responsible for their early deaths.

The early complication rate was low (1%), and the intraoperative handling was excellent. We further analysed early patch-related reoperations/stent implants after an initial patch implant within the entire study population and found acceptable results (4.9% of all patients/8.9% of all locations). Similar encouraging data were reported in a recent study on short-term results (mean follow-up = 12 months) comparing decellularized equine pericardium to bovine pericardium when used in different locations in congenital heart surgery [21].

With the goal of shedding light on midterm patch durability, we focused only on survivors with intact patch locations at the time of discharge and those with a follow-up duration of ≥ 12 months. A median follow-up duration of almost 30 months, a fair sample size of 132 patients and 200 locations and an overall data-completeness of >95% are comparable to the results from most other studies focusing on midterm patch durability [4, 21].

We reported a total of 15.5% late catheter reinterventions (31/200) and 11% late patch-related reoperations (22/200) per location with 12- and 24-months overall freedom from reoperation/reintervention per location of 91.5% and 85.2%, respectively, with the majority occurring after PA augmentation (88.7%). These results match the findings of our binary logistic regression model (OR for PA implantation site = 2.44) as well as the freedom from reoperation/catheter reintervention after a patch implant in the PA position (85.1%) with other locations (98.9%). We found a significant difference (*P*<0.001) between the 2 implant locations, which suggests that patients after PA augmentation are at higher risk for reoperation/reintervention.

When we reviewed the literature for experiences with other xenogeneic patch materials, we found diverse results. Baird *et al.* described their institutional experience with photo-oxidized bovine pericardium with lower early reoperation (1%)/reintervention rates (1%) and similar late reoperation (4.4%)/reintervention rates (8%). However, their follow-up duration with a mean of 20 months was rather short to rate midterm patch durability. Furthermore, they described patch-related reoperations/reinterventions per patient and not per location, as we did [3].

On the other hand, Gluck *et al.* reported similar reoperation (12/134 patients, 9%) but higher reintervention rates (75/134, 56%) after glutaraldehyde-treated cryopreserved homograft pericardium implants with a median follow-up duration of 5.3 years. Again, this group calculated patch-related reoperations/reinterventions per patient, not per location [5].

Finally, Bell *et al.* reported a total reintervention/reoperation rate as low as 3.6% (n=18) per 501 tissue-engineered bovine pericardium patch implants in a median follow-up time of 30 months [22].

Even though our current experience with decellularized equine pericardium showed relatively high reintervention/reoperation rates when used in the PA position, we have not yet changed our surgical strategy. We still feel that decellularized equine pericardium is a valuable option when autologous pericardium is not available. We also appreciate its pliable structure and excellent intraoperative handling. To adequately answer the question if decellularized equine pericardium is superior, inferior or equal to other xenogeneic patch materials when it comes to long-term durability, further prospective, comparative multicentre studies are needed.

### Limitations

This study has several limitations. First, because of the retrospective design of the study, our single-centre experience and the lack of a control, there is certainly the potential for confounding/selection bias. Because we were able to show our own experiences with this patch, generalization of our results must be made with caution. Judgements of related to intraoperative handling can be dependent on the surgical experience in the individual centre. Secondly, as described earlier, we recently started to use decellularized equine pericardium in other implant locations besides the PAs. Its use in systemic arterial and/or valvular locations was restrictive, which results in relatively small numbers of other locations and makes a fair comparison difficult. We tried to correct for these confounders by using a binary logistic regression model to analyse risk factors for patch-related reoperations/reinterventions. However, due to the small numbers of other implant locations as well as the small number of events, the results must be treated with caution. Longer follow-up durations are certainly needed to draw conclusions regarding long-term durability. Finally, the fact that we needed to exclude a fair number of patients (n=49) with a follow-up period < 12 months, could create another possible selection bias, i.e. we missed potential patch-related reoperations/reinterventions in patients who were lost to follow-up but may have been treated in other centres. On the other hand, we believe that patients with longer follow-up are more representative of midterm patch performance.

### Conclusion

Nevertheless, our data indicate that decellularized equine pericardium can be used to repair a variety of congenital heart defects with satisfying early performance and acceptable midterm durability. Reoperation/reintervention rates were in a range similar to those observed with other reported xenogeneic materials in the literature, with the majority occurring after PA augmentation. The search for an ideal patch material for repair of congenital heart defects surely remains an ongoing challenge. Prospective well-powered studies based on multicentre registries are urgently needed to analyse long-term patch durability.

## Data Availability

Raw data were generated at the German Heart Center Berlin. Derived data supporting the findings of the study are available from the corresponding author (PM) on request.

## Notes

Meeting presentation: 51st Annual Meeting of the German Society for Thoracic and Cardiovascular Surgery, February 2022

## ABBREVIATIONS

IQR: Interquartile range
PA: Pulmonary artery
STAT score/category: Society of Thoracic Surgeons–European Association for Cardio-thoracic Surgery) mortality risk score/category
